# Association between the triglyceride-glucose index and in-hospital major adverse cardiovascular events in patients with acute coronary syndrome: results from the Improving Care for Cardiovascular Disease in China (CCC)-Acute Coronary Syndrome project

**DOI:** 10.1186/s12933-024-02270-7

**Published:** 2024-05-15

**Authors:** Wenjie Wang, Jiaxin Yang, Kexin Wang, Jialong Niu, Yixuan Liu, Hailong Ge, CCC-ACS Investigators

**Affiliations:** Department of Cardiology, Beijing Anzhen Hospital, Capital Medical University, Anzhen Avenue #2, Chaoyang District, 100029 Beijing, People’s Republic of China

## Abstract

**Objective:**

Although the TyG index is a reliable predictor of insulin resistance (IR) and cardiovascular disease, its effectiveness in predicting major adverse cardiac events in hospitalized acute coronary syndrome (ACS) patients has not been validated in large-scale studies. In this study, we aimed to explore the association between the TyG index and the occurrence of MACEs during hospitalization.

**Methods:**

We recruited ACS patients from the CCC-ACS (Improving Cardiovascular Care in China-ACS) database and calculated the TyG index using the formula ln(fasting triglyceride [mg/dL] × fasting glucose [mg/dL]/2). These patients were classified into four groups based on quartiles of the TyG index. The primary endpoint was the occurrence of MACEs during hospitalization, encompassing all-cause mortality, cardiac arrest, myocardial infarction (MI), and stroke. We performed Cox proportional hazards regression analysis to clarify the correlation between the TyG index and the risk of in-hospital MACEs among patients diagnosed with ACS. Additionally, we explored this relationship across various subgroups.

**Results:**

A total of 101,113 patients were ultimately included, and 2759 in-hospital MACEs were recorded, with 1554 (49.1%) cases of all-cause mortality, 601 (21.8%) cases of cardiac arrest, 251 (9.1%) cases of MI, and 353 (12.8%) cases of stroke. After adjusting for confounders, patients in TyG index quartile groups 3 and 4 showed increased risks of in-hospital MACEs compared to those in quartile group 1 [HR = 1.253, 95% CI 1.121–1.400 and HR = 1.604, 95% CI 1.437–1.791, respectively; *p* value for trend < 0.001], especially in patients with STEMI or renal insufficiency. Moreover, we found interactions between the TyG index and age, sex, diabetes status, renal insufficiency status, and previous PCI (all *p* values for interactions < 0.05).

**Conclusions:**

In patients with ACS, the TyG index was an independent predictor of in-hospital MACEs. Special vigilance should be exercised in females, elderly individuals, and patients with renal insufficiency.

**Supplementary Information:**

The online version contains supplementary material available at 10.1186/s12933-024-02270-7.

## Introduction

Cardiovascular disease (CVD) remains the leading cause of death worldwide, with an estimated 17.9 million fatalities annually. Among these, acute coronary syndrome (ACS) is a significant contributor to CVD-related mortality [1]. ACS encompasses a cluster of conditions characterized by impaired myocardial function or necrosis due to diminished blood flow in the coronary arteries [2].

Insulin resistance (IR) is characterized by insulin sensitivity impairment, as demonstrated by a shift towards higher insulin concentrations on the insulin concentration-effect curve [3]. At elevated plasma insulin concentrations, IR is a condition in which insulin-target tissues do not adequately dispose of blood glucose, suppress endocrine glucose production (EGP), and stimulate glycogen production [3]. Recent studies have demonstrated that IR is independent of the onset and progression of CVD and chronic kidney disease (CKD) [4]. According to a study conducted on patients referred for coronary angiography, only a predisposition to IR (i.e., a high genetic risk score burden) was associated with coronary artery disease risk [5]. Another study revealed that compared with hypertension, obesity, and smoking, IR appears to be the strongest risk factor for premature onset of coronary heart disease [6]. Although the hyperinsulinaemic-euglycaemic clamp (HIEC) represents the gold standard for measuring insulin resistance, this technique is difficult to perform in clinical practice because it involves several difficult procedures [7]. In recent years, TyG index has gained increasing popularity as alternative methods for diagnosing IR [8–10]. Studies have demonstrated that there is an association between the TyG index and heart failure [11], coronary heart disease [12] and the development of hypertension [13].

The main risk factors associated with ACS include advanced age, smoking, diabetes, hyperlipidaemia, hypertension, and increased body mass index (BMI) [14]. According to some studies, the TyG index is associated with ACS occurrence and prognosis [15]. Currently, large-scale studies exploring the correlation between the TyG index and the occurrence of MACEs during hospitalization in patients with ACS are lacking, leaving this association unclear. Therefore, the purpose of this study was to explore relationships among these factors, assisting clinicians in determining the risk of in-hospital MACE occurrence in patients and reducing mortality rates.

## Methods

### Study design and data sources

A national registry and quality improvement study, CCC-ACS (Improving Cardiovascular Care in China-ACS), is a database of focusing on the quality of ACS care in China. The American Heart Association and the Chinese Society of Cardiology initiated this study in 2014. The CCC-ACS [16] was designed and implemented using Oracle Clinical Remote Data Capture, an industry standard data collection platform. The participants’ hospitals received data from an abstractor trained to extract the required information from medical records. The CCC-ACS database was continuously updated by the middle of the following month for all eligible patients. A third-party clinical research associate was hired to conduct a quality audit of the case reports to ensure that they were continuous and not selective. Additionally, 5% of the reported cases were randomly selected from each participating centre. After selecting the data, they were compared with the original medical records to ensure accuracy and completeness. However, the quality audit report indicated that the data were properly reported, despite the low number of missing data and errors.

**Fig. 1:**
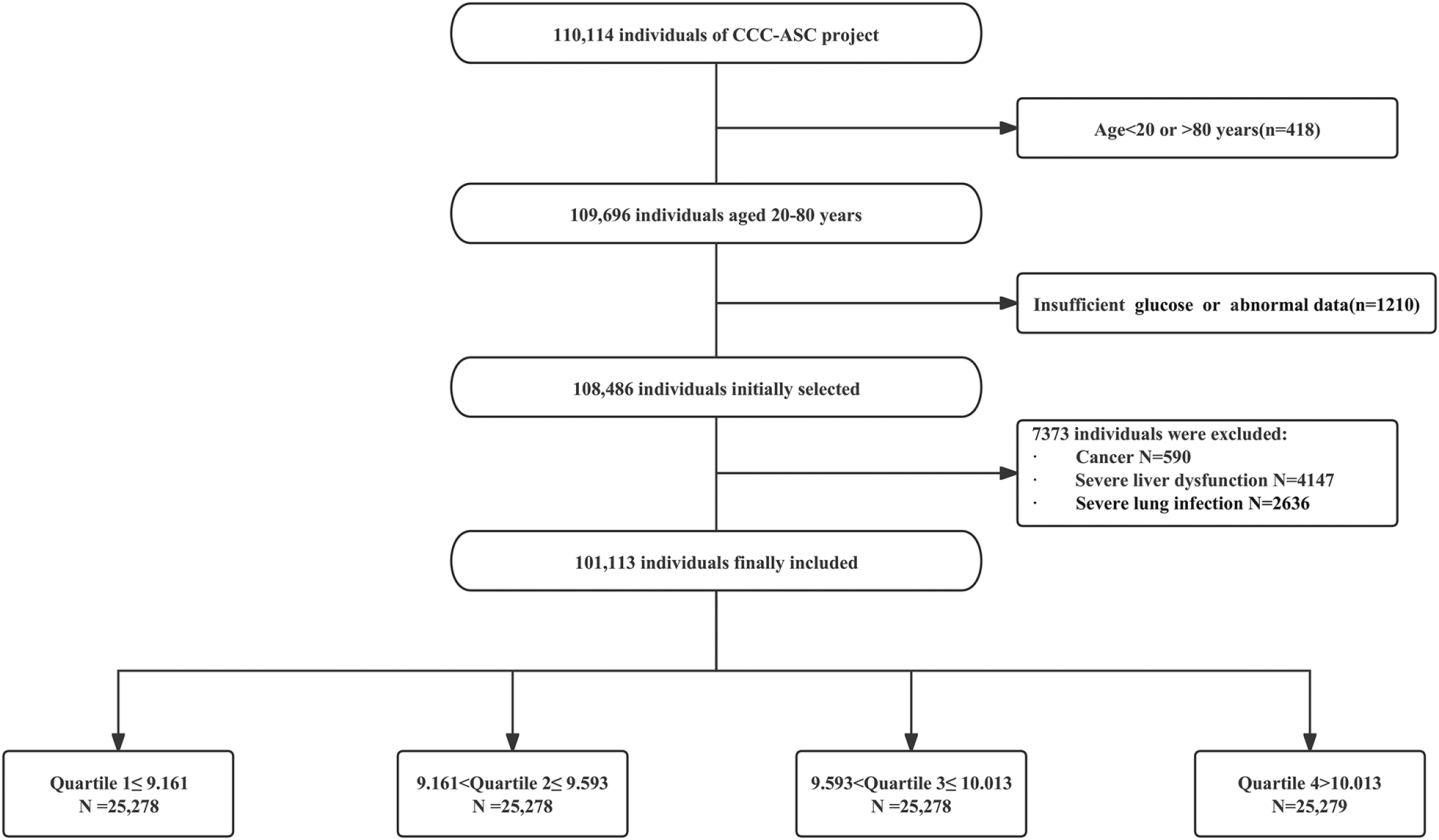
Flow chart of enrolment

The data of 110,114 patients with ACS were collected from November 2014 to December 2019 in 240 hospitals registered with the ACS registry. After excluding patients aged < 20 years or > 80 years (n = 418 patients), patients with missing or abnormal data (1210 patients), patients with tumours (590 patients), patients with severe hepatic insufficiency (4147 patients), and patients with pulmonary infections (2636 patients), a total of 101,113 patients were ultimately included in this study (Fig. 1). The study was approved by a research ethics committee with a wavier of patient consent, and strictly adhered to the Declaration of Helsinki.

### Data collection and definition

Records of hypertension and hyperlipidaemia were obtained from the patient’s previous medical history. On the second day after hospital admission, fasting blood samples were collected to measure creatinine levels, lipid profiles, and blood glucose levels. The TyG index was calculated according to the formula [ln(fasting triglycerides [mg/dL] × fasting glucose [mg/dL]/2) [17]. Based on the Modification of Diet in Renal Diseases equation for Chinese patients, the baseline eGFR was calculated: eGFR (mL/min/1.73 m^2^) = 175 × SCr (mg/dl)^−1.234^ × Age^−0.179^ (× 0.79 for women) [18]. The primary endpoint was in-hospital MACEs, which included a composite of all-cause mortality, cardiac arrest, cardiogenic shock, myocardial infarction (MI), and stroke. The secondary endpoints were all-cause death, cardiac arrest, cardiogenic shock, MI, and ischaemic stroke.

### Statistical analysis

The included subjects were stratified according to TyG index quartile. The means and standard deviations of normally distributed continuous variables are shown, while medians and interquartile ranges are shown for nonnormally distributed variables. Categorical variables are presented as number (percentage). For continuous variables, the Kruskal–Wallis test was used, whereas the χ2 test or Fisher’s exact probability test was used for categorical variables.

The Cox proportional hazards model was used to investigate the association between the TyG index and MACEs during hospitalization. Model 1 was unadjusted, Model 2 was adjusted for age and sex, and Model 3 was further adjusted for variables with *p* < 0.1 in the univariate analysis, including left ventricular ejection fraction (LVEF), haemoglobin A1C (HbA1C), smoking status, hypertension status, hyperlipidaemia status, diabetes status, previous cerebrovascular disease, previous peripheral vascular disease, previous myocardial infarction (MI), previous chronic obstructive pulmonary disease, history of valve surgery, oral aspirin, oral statins, oral clopidogrel, oral hypoglycaemic agents, and subcutaneous insulin. Next, we also conducted stratified analysis based on different diagnoses, including unstable angina (UA), non-ST-segment elevation myocardial infarction (NSTEMI) and ST-segment elevation myocardial infarction (STEMI). Additionally, the patients were further analysed in subgroups according to age, sex, smoking status, diabetes status, hyperlipidaemia status, renal function, hypertension status, and previous percutaneous coronary intervention (PCI). Finally, we divided the patients into 5 groups based on the estimated glomerular filtration rate (eGFR) to examine the relationship between the TyG index and MACEs across various levels of renal function. Two-tailed *p* values less than 0.05 was considered to indicate statistical significance. We used R 4.1.3 (R Foundation for Statistical Computing, Vienna, Austria) and IBM SPSS Statistics 26.0 (IBM Corporation, Chicago, IL) to perform the statistical analyses.

## Results

### Baseline characteristics of the study participants

A total of 101,113 patients were eventually enrolled in the study. According to the quartile distribution of the TyG index, patients were divided into four groups (Quartile 1: n = 25,278, TyG index ≤ 9.161; Quartile 2: n = 25,278, 9.161 < TyG index ≤ 9.593; Quartile 3, n = 25,278, 9.593 < TyG index ≤ 10.013. Quartile 4: n = 25,279, TyG index > 10.013) (Table 1). From Quartile 1 to Quartile 4, patient age, male proportion, total cholesterol (TC), low-density lipoprotein cholesterol (LDL-C), triglycerides (TG), and fasting blood glucose (FBG) gradually decreased, while high-density lipoprotein cholesterol (HDL-C) and the proportions of diabetes, hypertension, hyperlipidaemia, and renal insufficiency gradually increased (*P* value for trend < 0.05). Similarly, the proportions of patients receiving oral angiotensin II receptor blockers (ARBs), glucose-lowering medication, and subcutaneous insulin also increased gradually with quartile grouping (*P* value for trend < 0.05). However, the proportions of previous PCI (2263, 9.0%), previous MI (2336, 9.2%), and previous stroke (2212, 8.8%) were higher in group Quartile 1 than in the other three quartile groups. Significant statistical differences were observed among the four groups in multi-vessel CAD status, LVEF, and statin use (*P* value for trend < 0.05).

**Table 1:** Baseline characteristics according to quartiles of the TyG index

Variable	Quartile 1 (≤ 9.161)	Quartile 2 (9.161–9.593)	Quartile 3 (9.593–10.013)	Quartile 4 (> 10.013)	*P*-value
*Demographics*					
Age	67 (58,75)	64 (56, 73)	63 (54, 71)	61 (52, 69)	< 0.001
Male	18,972 (75.1%)	18,673 (73.9%)	18,311 (72.4%)	18,065 (71.5%)	< 0.001
*Risk factors*					
Diabetes (n (%))	2519 (10.0%)	3994 (15.8%)	6312 (24.9%)	10,033 (39.7%)	< 0.001
Hypertension (n (%))	11,888 (47%)	13,478 (53.3%)	14,155 (56.0%)	14,595 (57.7%)	< 0.001
Hyperlipidemia (n (%))	7696 (30.4%)	11,674 (46.2%)	19,043 (75.3%)	23,711 (92.1%)	< 0.001
Renal insufficiency (n (%))	2261 (8.9%)	2261 (8.9%)	2535 (10.0%)	2946 (11.7%)	< 0.001
Smoking (n (%))	6148 (24.3%)	6271 (25.8%)	6175 (24.4%)	6186 (24.4%)	0.189
*Basic medical history*					
Previous MI (n (%))	2263 (9.0%)	2134 (8.4%)	2109 (8.3%)	2024 (8.0%)	< 0.001
Previous stroke (n (%))	2336 (9.2%)	2307 (9.1%)	2235 (8.8%)	2037 (8.1%)	< 0.001
Previous PCI (n (%))	2212 (8.8%)	2205 (8.7%)	2192 (8.7%)	2143 (8.5%)	< 0.001
*Clinical presentations*					
Multi-vessel CAD (n (%))	1862 (7.4%)	2123 (8.4%)	2313 (9.2%)	2273 (9.0%)	< 0.001
LVEF	53 (37, 61)	54 (37, 61)	54 (36, 61)	53 (30, 61)	< 0.001
*Laboratory measurements*					
TC (mmol/l)	3.98 (3.35, 4.69)	4.29 (3.61, 5.02)	4.51 (3.78, 5.27)	4.76 (3.93, 5.58)	< 0.001
LDL-C (mmol/l)	2.37 (1.84, 2.97)	2.64 (2.07, 3.26)	2.78 (2.18, 3.41)	2.83 (2.2, 3.5)	< 0.001
HDL-C (mmol/l)	1.13 (0.94, 1.35)	1.06 (0.89, 1.27)	1.02 (0.86, 1.22)	0.98 (0.82, 1.19)	< 0.001
Triglycerides (mmol/l)	0.84 (0.67, 1)	1.29 (1.09, 1.5)	1.78 (1.43, 2.15)	2.83 (2.2, 3.5)	< 0.001
FPG (mmol/l)	5.10 (4.55, 5.94)	5.64 (5.60, 6.67)	6.4 (5.40, 8.04)	8.43 (6.42, 11.24)	< 0.001
Creatinine	80 (67, 98)	80 (67, 97)	80 (67, 98)	80 (66, 100)	0.597
*Medications at discharge*					
Statins (n (%))	4406 (17.4%)	4709 (18.6%)	4668 (18.5%)	4423 (17.5%)	0.003
ACEIs (n (%))	1292 (5.1%)	1340 (5.3%)	1326 (5.2%)	1379 (5.5%)	0.747
ARBs (n (%))	4803 (19.0%)	5340 (21.1%)	5575 (22.1%)	5741 (22.7%)	< 0.001
Insulin (n (%))	659 (2.6%)	1003 (4.0%)	1673 (6.6%)	2673 (10.6%)	< 0.001
Oral hypoglycemic agents (n (%))	1421 (5.6%)	2286 (9.0%)	3603 (14.3%)	5674 (22.4%)	< 0.001

MI, myocardial infarction; PCI, percutaneous coronary intervention; CAD, coronary artery disease; LVEF, left ventricular ejection fraction; TC, total cholesterol; LDL-C, low-density lipoprotein-cholesterol; HDL-C, high-density lipoprotein-cholesterol; FPG, fasting plasma glucose; ACEIs, angiotensin-converting enzyme inhibitors; ARBs, angiotensin receptor blockers

**Fig. 2:**
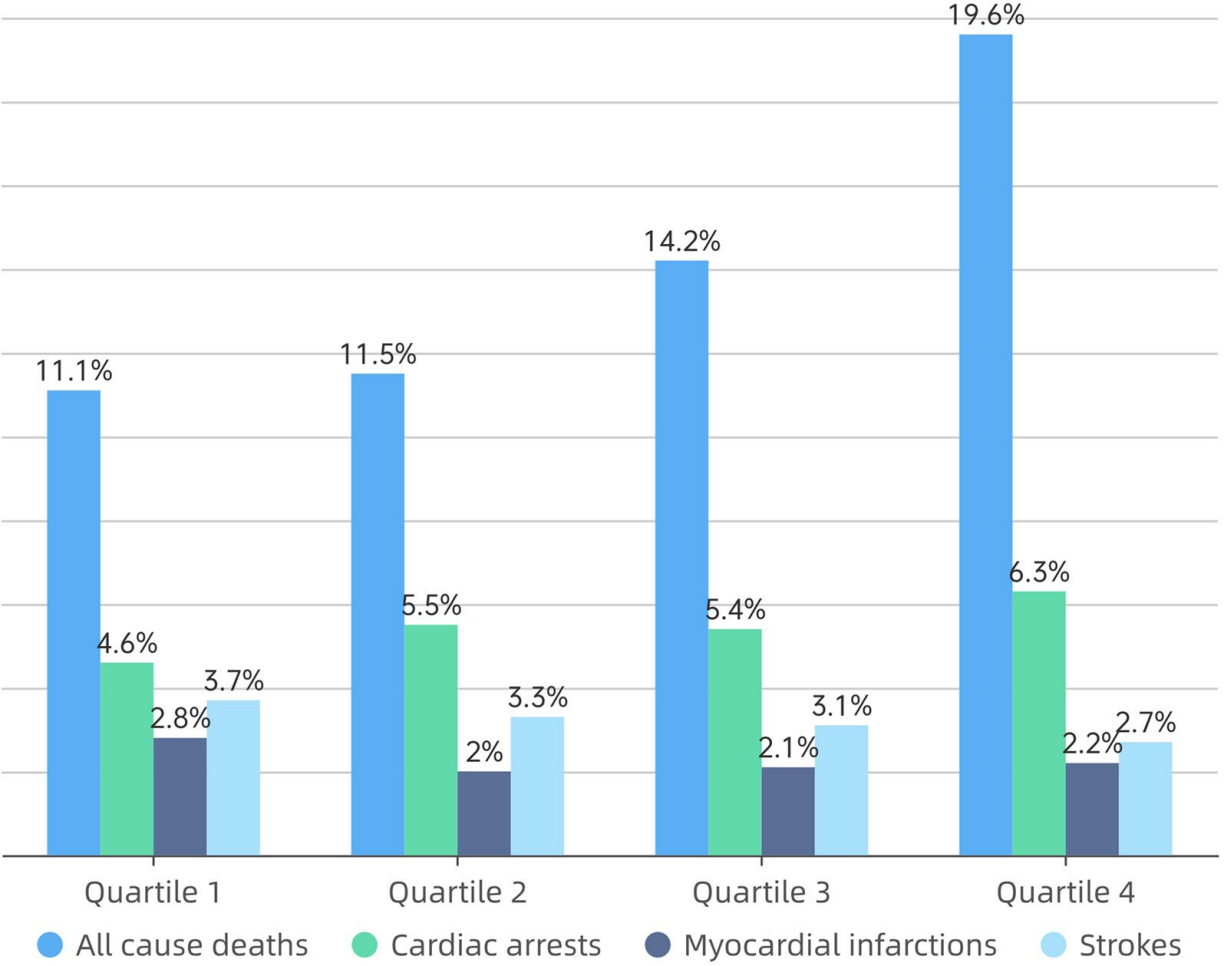
Incidence of MACEs patients with ACS under different TyG index

In this study, 2759 MACEs were recorded, of which 1554 (49.1%) were all-cause mortality, 601 (21.8%) were cardiac arrests, 251 (9.1%) were MI and 353 (12.8%) were strokes. From Quartile 1 to Quartile 4, as the TyG index gradually increased, the incidence of MACEs also increased progressively, which were 609 (22.1%), 614 (22.3%), 685 (24.8%) and 851 (30.8%), respectively. The proportions of each endpoint of MACE across different groups are illustrated in Fig. 2.

### Analysis of the TyG index and in-hospital MACEs

Cox regression analysis was conducted on the ACS patients to evaluate the association between the TyG index and MACEs. According to the univariate Cox regression analysis, there was no significant association between the TyG index and MACEs in patients with ACS. After adjusting for confounders (Table 2), there was an increased risk of in-hospital MACEs in Quartile 3 [HR = 1.253, 95% CI 1.121–1.4] and Quartile 4 [HR = 1.604, 95% CI 1.437–1.791] in comparison with Quartile 1(*P* value for trend < 0.001). Among patients with UA, an elevated TyG index did not increase the risk of in-hospital MACEs (Table 3). The risk of in-hospital MACEs increased with increasing TyG index in patients with NSTEMI and STEMI. After adjusting for Model 2, significantly increased risks of in-hospital MACEs in patients with NSTEMI were observed in Quartile 3 [HR = 1.307, 95% CI 1.046–1.634] and Quartile 4 [HR = 1.715, 95% CI 1.382–2.217] compared to Quartile 1, as well as in Quartile 4 [HR = 1.558, 95% CI 1.243–1.954] after adjusting for Model 3. In STEMI patients, whether adjusted for confounders or not, the risk of MACEs in Quartiles 3 and 4 was significantly greater than that in Quartile 1, especially in Quartiles 3 [HR = 1.276, 95% CI 1.118–1.458] and 4 [HR = 1.67, 95% CI 1.464–1.904] in Model 3.

**Table 2:** Associations between TyG index and MACEs

TyG indexes	HR (95% CI)		
	Model 1	Model 2	Model 3
Quartile 1 (≤ 9.161)	Reference	Reference	Reference
Quartile 2 (9.161–9.593)	0.986 (0.803, 1.209)	1.105 (0.987, 1.236)	1.098 (0.981, 1.229)
Quartile 3 (9.593–10.013)	1.033 (0.843, 1.265)	1.304 (1.168, 1.456)	1.253 (1.121, 1.4)
Quartile 4 (> 10.013)	1.085 (0.889, 1.325)	1.76 (1.583, 1.956)	1.604 (1.437, 1.791)
*P* value for trend	0.786	< 0.001	< 0.001

Model 1. Original data

Model 2. Minimally adjusted models were adjusted for age and sex

Model 3. Multivariable adjusted models were additionally adjusted for left ventricular ejection fraction, Hemoglobin A1C, smoking, hypertension, hyperlipidemia, diabetes, previous cerebrovascular disease, previous peripheral vascular disease, previous myocardial infarction, previous chronic obstructive pulmonary disease, history of valve surgery, oral aspirin, oral statins, oral clopidogrel, oral hypoglycemic agents, and subcutaneous insulin

**Table 3:** Associations between TyG index and MACEs in UAP, NSTEMI and STEMI

TyG indexes	HR (95% CI)								
	UA			NSTEMI			STEMI		
	Model 1	Model 2	Model 3	Model 1	Model 2	Model 3	Model 1	Model 2	Model 3
Quartile 1	Reference	Reference	Reference	Reference	Reference	Reference	Reference	Reference	Reference
Quartile 2	0.86 (0.526, 1.408)	0.88 (0.543, 1.426)	0.88 (0.542, 1.43)	0.944 (0.739, 1.205)	1.070 (0.851, 1.345)	1.082 (0.860, 1.361)	0.977 (0.836, 1.141)	1.142 (0.998, 1.305)	1.114 (0.974, 1.274)
Quartile 3	0.821 (0.497, 1.358)	0.951(0.588, 1.539)	0.93(0.571, 1.516)	1.043 (0.822, 1.323)	1.307 (1.046, 1.634)	1.240 (0.990, 1.554)	1.179 (1.016,1.367)	1.346 (1.18, 1.535)	1.276 (1.118, 1.458)
Quartile 4	0.674 (0.392, 1.158)	0.828 (0.496, 1.382)	0.761 (0.446, 1.298)	1.329 (1.061, 1.665)	1.715 (1.382, 2.127)	1.558 (1.243, 1.954)	1.527 (1.327, 1.757)	1.89 (1.666, 2.145)	1.670 (1.464, 1.904)
*P* value for trend	0.554	0.892	0.784	< 0.001	< 0.001	0.001	< 0.001	< 0.001	< 0.001

Model 1. Original data

Model 2. Minimally adjusted models were adjusted for age and sex

Model 3. Multivariable adjusted models were additionally adjusted for left ventricular ejection fraction, Hemoglobin A1C, smoking, hypertension, hyperlipidemia, diabetes, previous cerebrovascular disease, previous peripheral vascular disease, previous myocardial infarction, previous chronic obstructive pulmonary disease, history of valve surgery, oral aspirin, oral statins, oral clopidogrel, oral hypoglycemic agents, and subcutaneous insulin

### TyG index and subgroup analysis

**Fig. 3:**
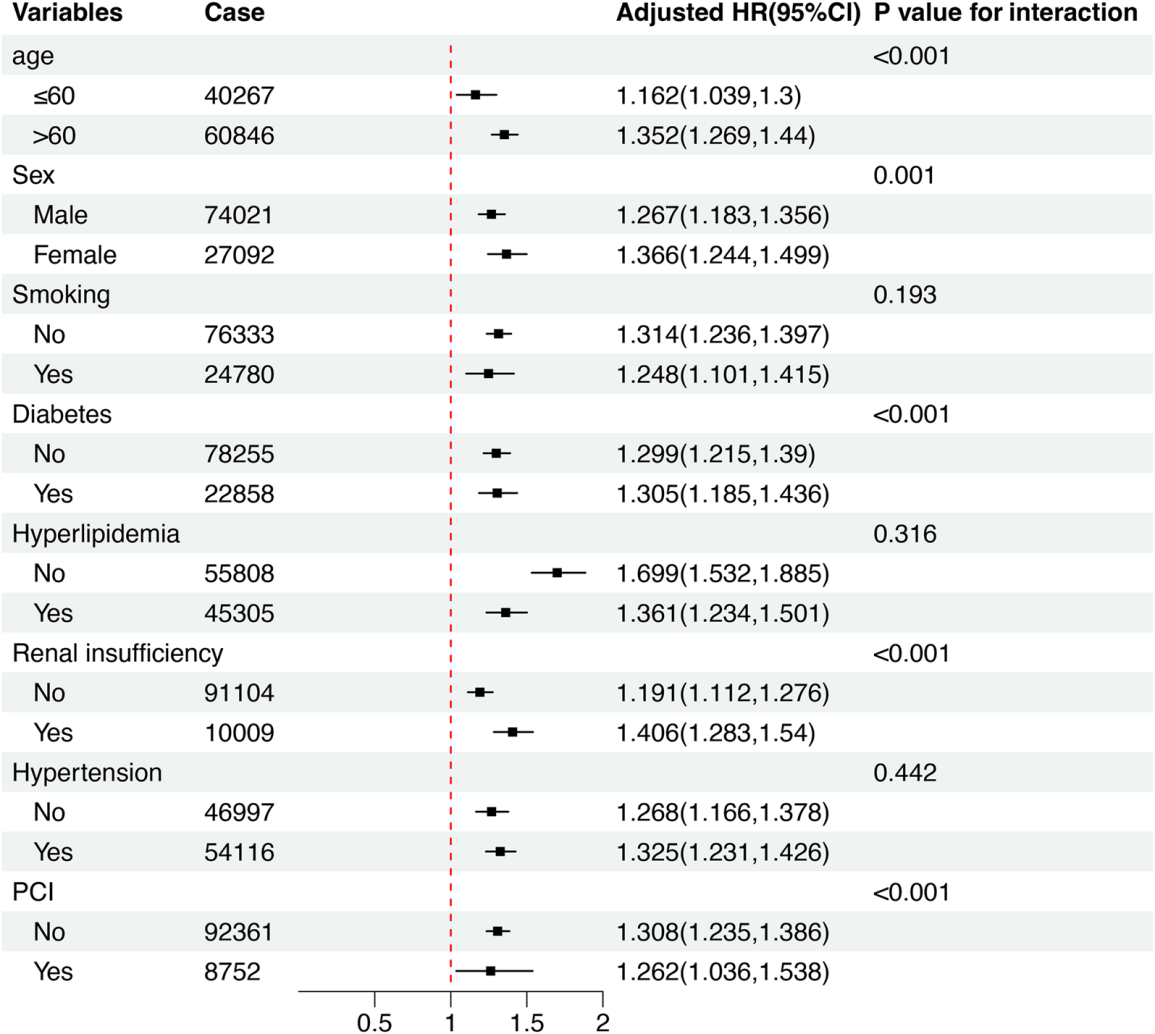
Joint association of TyG index and MACEs; TyG index were classified into 4 groups: ≤ 9.161, 9.161–9.593, 9.593–10.013, > 10.013,Models corrected for sex, age, cerebrovascular disease, previous MI, smoking status, statins, previous hypertension, history of valve surgery, peripheral vascular disease,HbA1C, diabetes, chronic obstructive pulmonary disease, oral aspirin or not, oral clopidogrel or not, history of angina, creatinine, Left ventricular ejection fraction, subcutaneous insulin or not

To further explore the relationship between the TyG index and in-hospital MACEs, we conducted subgroup analyses. In each subgroup, the TyG index was significantly associated with an increased risk of in-hospital MACEs after adjusting for confounders, which confirmed the robustness of the results (Fig. 3). Moreover, we found interactions between the TyG index and age, sex, diabetes status, renal insufficiency status, and previous PCI with the TyG index (all *p* values for interactions < 0.05).

### Associations between the TyG index and MACEs for different eGFRs

To further explore the relationships among different renal function levels, we categorized patients into five groups based on eGFR. In patients with normal renal function (eGFR ≥ 90 ml/min/1.73 m^2^), Quartile 4 [HR = 1.339, 95% CI 1.086–1.651] remained associated with an increased risk of in-hospital MACEs after adjusting for confounders (Table 4). The risk of MACEs was significantly greater in Quartile 4 [HR = 1.339, 95% CI 1.086–1.651] with 90 > eGFR ≥ 60 ml/min/1.73 m^2^, Quartile 3 [HR = 1.47, 95% CI 1.076–2.01] and Quartile 4 [HR = 1.793, 95% CI 1.312–2.449] with 60 > eGFR ≥ 45 ml/min/1.73 m^2^; Quartile 4 [HR = 1.98, 95% CI 1.472–2.663] with 45 > eGFR ≥ 30 ml/min/1.73 m^2^, and Quartile 3 [HR = 1.418, 95% CI 1.079–1.864] and Quartile 4 [HR = 1.793, 95% CI 1.372–2.342] with eGFR < 30 ml/min/1.73 m^2^.

**Table 4:** Associations between TyG index and MACEs in different eGFR

Variables	Case	Unadjusted HR (95% Cl)	Adjusted HR (95% Cl)	*P* value	*P* value for trend
eGFR ≥ 90 ml/min·1.73 m^2^	51,266				0.006
Quartile 1		Reference	Reference		
Quartile 2		0.986 (0.803, 1.209)	0.951 (0.772, 1.17)	0.634	
Quartile 3		1.033 (0.843, 1.265)	1.039 (0.843, 1.281)	0.719	
Quartile 4		1.085 (0.889, 1.325)	1.339 (1.086, 1.651)	0.006	
90 > eGFR ≥ 60 ml/min·1.73 m^2^	32,271				0.006
Quartile 1		Reference	Reference		
Quartile 2		0.908 (0.739, 1.117)	0.951 (0.772, 1.171)	0.635	
Quartile 3		0.966 (0.787, 1.186)	1.04 (0.843, 1.282)	0.716	
Quartile 4		1.263 (1.038, 1.537)	1.339 (1.086, 1.651)	0.006	
60 > eGFR ≥ 45 ml/min·1.73 m^2^	8360				0.003
Quartile 1		Reference	Reference		
Quartile 2		1.211 (0.883, 1.661)	1.3 (0.945, 1.789)	0.106	
Quartile 3		1.384 (1.019, 1.88)	1.47 (1.076, 2.01)	0.016	
Quartile 4		1.645 (1.226, 2.208)	1.793 (1.312, 2.449)	< 0.001	
45 > eGFR ≥ 30 ml/min·1.73 m^2^	5239				< 0.001
Quartile 1		Reference	Reference		
Quartile 2		1.175 (0.859, 1.606)	1.296 (0.945, 1.777)	0.108	
Quartile 3		1.117 (0.822, 1.518)	1.253 (0.916, 1.712)	0.158	
Quartile 4		1.621 (1.223, 2.148)	1.98 (1.472, 2.663)	< 0.001	
eGFR < 30 ml/min·1.73 m^2^	3977				< 0.001
Quartile 1		Reference	Reference		
Quartile 2		1.114 (0.833, 1.49)	1.113 (0.831, 1.491)	0.473	
Quartile 3		1.419 (1.084, 1.858)	1.418 (1.079, 1.864)	0.012	
Quartile 4		1.637 (1.268, 2.113)	1.793 (1.372, 2.342)	< 0.001	

Model 1. Original data

Model 2 Adjusted models were adjusted for age, sex, left ventricular ejection fraction, Hemoglobin A1C, smoking, hypertension, hyperlipidemia, diabetes, previous cerebrovascular disease, previous peripheral vascular disease, previous myocardial infarction, previous chronic obstructive pulmonary disease, history of valve surgery, oral aspirin, oral statins, oral clopidogrel, oral hypoglycemic agents, and subcutaneous insulin

## Discussion

This large-scale study to investigate the relationship between the TyG index and in-hospital MACE risk in ACS patients. The results indicated that the incidence and risk of MACEs gradually increase with increasing TyG index, particularly among patients diagnosed with STEMI. Furthermore, we demonstrated the robustness of the results in different subgroups and found interactions between the TyG index and age, sex, diabetes status, renal insufficiency status, and previous PCI.

As a surrogate marker for insulin resistance, the TyG index has now been introduced. As a marker of vascular endothelial inflammation and functional damage [19, 20], the TyG index is strongly associated with the risk of atherosclerosis and cardiovascular disease [12]. Wu et al. [17] randomly selected 6095 patients who did not have diabetes or cardiovascular disease and who were followed for 10 years and found that increases in the TyG index quartile were associated with a greater prevalence of CVD. Insulin is able to reduce the synthesis of VLDL under normal physiological conditions by activating PI3K, which degrades apoB. Insulin resistance, on the other hand, inhibits this degradation, which leads to an increase in the production of VLDL [21].The presence of insulin resistance can result in systemic lipid disturbances, including elevated levels of total cholesterol, small dense lipoproteins (LDL), and postprandial lipids, and reduced levels of high-density lipoprotein (HDL), which can contribute to the onset of atherosclerosis [22, 23]. Moreover, reduced insulin activity in established ischaemic myocardium impairs glucose bioavailability and alters fatty acid metabolism, leading to increased oxygen consumption and decreased compensatory capacity in noninfarcted myocardium [24]. These pathological metabolic disturbances further exacerbate the progression of CAD. We found that the risk of in-hospital MACEs increased as the TyG index increased after adjusting for relevant factors in our study. Wang et al. [25] studied 935 patients with ACS and reported that as the severity of CAD increased with increasing TyG index, the incidence of MACEs also increased, which is consistent with the results of our study. In addition we found that the incidence of in-hospital MACEs increased with increasing TyG index in patients with STEMI and NSEMI. But there was no significant relationship in patients with unstable angina. The mechanism underlying the association between patients with ACS and TyG index is currently unclear, but this correlation may be based on the state of insulin resistance as assessed by the TyG index. Hyperglycaemia caused by insulin resistance can lead to an increase in reactive oxygen species and inflammatory factors, which can cause endothelial dysfunction [26]. The relationship between acute MI and IR may be explained by localized platelet activation and thrombin generation, as well as an increase in coronary thrombotic load, as a result of acute MI [27, 28]. However, the mechanism of unstable angina and in-hospital MACEs is currently mechanistically unclear and needs to be further explored.

Subgroup analyses revealed that patients with ACS in renal insufficiency had a greater likelihood of experiencing in-hospital MACEs and the TyG index of patients with mild to moderate renal insufficiency was strongly related to MACEs in our study. This may be due to metabolic acidosis, intestinal dysregulation, increased chronic inflammation activation, or uraemic toxins which exacerbate insulin resistance in patients with ACS during chronic renal failure [29–32]. Several mechanisms contribute to insulin resistance, which in turn causes endothelial damage and atherosclerosis in coronary arteries [33], thereby causing in-hospital MACEs.

In our study, we found that the risk of in-hospital MACEs was greater in diabetic patients than in nondiabetic patients. Wang et al. [15] also verified that the TyG index predicted future MACEs in patients with diabetes and ACS independently of known cardiovascular risk factors, suggesting that the TyG index may be a useful marker for risk stratification and prognosis in patients with diabetes and ACS. The largest number of patients with previous PCI, previous MI, and previous stroke were in Quartile 1, possibly because people with previous illnesses pay more attention to their blood glucose and blood lipids. We found that the risk of in-hospital MACEs was greater among female ACS patients than among males patients in subgroups by sex. Lu et al. [22] reported that a greater TyG index was associated with the development of subclinical atherosclerosis in nondiabetic female patients, but not observed in nondiabetic male patients. Compared with men,women were reported to be at higher risk of MI when they have a high TyG index [33]. A cohort study revealed that type 1 diabetes affects adiposity and skeletal muscle insulin sensitivity more strongly in women than in men, which may contribute to the relatively higher cardiovascular risk among women [34]. Arshi et al. [35] reported that high insulin levels and HOMA-IR were significantly associated with hypertension prevalence but not in men.

In our study, patients with hyperlipidaemia had a greater risk of MACEs as the TyG index increased. Hyperlipidaemia patients are often accompanied by increased TG and small dense LDL levels and lower high-density lipoprotein (HDL) levels, all of which exacerbate the occurrence of MACEs in ACS.

Additionally, due to the observational study design used in this analysis, we cannot exclude residual or unmeasured confounding even after adjusting for potential cardiac risk factors. Second, the study population was selected, but the results may be biased because we did not consider the type, frequency, or duration of use of medications. Third, in this trial, the follow-up period was short and only in-hospital MACEs were counted, which may have led to some error on the results. Fourth, most of our fasting plasma glucose and fasting triglyceride values were collected on the next day of admission, but a small amount of data were collected on fasting plasma glucose and lipids were collected on the day of admission. Also, environmental factors may have influenced our results. Fifth, although our study was a multicentre study, we gave the current results may not be applicable to other ethnic groups because all of the participants in our study were only Chinese.

## Conclusion

In patients with ACS, the incidence and risk of in-hospital MACEs increase with an increasing TyG index, indicating that the TyG index is an independent predictor of in-hospital MACEs. The TyG index should receive more attention in females, elderly individuals and patients with renal insufficiency. Clinicians may further assess the risk of in-hospital MACEs in patients using the TyG index, potentially reducing in-hospital mortality.

### Supplementary Information

Additional file 1.

## Data Availability

The datasets analyzed during the current study are not publicly available because of intellectual property rights, but are available from the Prof. Dong Zhao on reasonable request.

## Abbreviations

TyG index: Triglyceride-glucose index
IR: Insulin resistance
CVD: Cardiovascular diseases
MACEs: Major adverse cardiovascular events
ACS: Acute coronary syndrome
CKD: Chronic kidney disease
HIEC: High insulin-normal glucose clamp
CCC-ACS: Cardiovascular Care in China-Acute Coronary Syndrome project
HR: Hazard ratio
LDL: Low-dense lipoproteins
