# Supplementary material for: Association between the triglyceride-glucose index and in-hospital major adverse cardiovascular events in patients with acute coronary syndrome: results from the Improving Care for Cardiovascular Disease in China (CCC)-Acute Coronary Syndrome project
Source: Cardiovasc Diabetol. 2024 May 15;23:170. doi: 10.1186/s12933-024-02270-7 (PMC11097581; doi:10.1186/s12933-024-02270-7)
Supplement: Supplementary file 1 — Additional file 1. [file 12933_2024_2270_MOESM1_ESM.docx]

Table S1. Investigators of CCC-ACS project.

| **ID** | **Hospitals** | **Territories** | **Provinces** | **City** | **Investigator** |
| --- | --- | --- | --- | --- | --- |
| 1 | Shanxi Cardiovascular Hospital | Northern China | Shanxi | Taiyuan | Bao Li |
| 2 | Nanjing Drum Tower Hospital, The Affiliated Hospital of Nanjing University Medical School | Eastern China | Jiangsu | Nanjing | Biao Xu, Guangshu Han |
| 3 | Hainan General Hospital | Southern China | Hainan | Haikou | Bin Li |
| 4 | The Second Hospital of Jilin University | Northeast China | Jilin | Changchun | Bin Liu |
| 5 | The 2nd Affiliated Hospital of Harbin Medical University | Northeast China | Heilongjiang | Harbin | Bo Yu |
| 6 | The Ninth Hospital Affiliated to Shanghai Jiaotong University School of Medicine | Eastern China | Shanghai | Shanghai | Changqian Wang |
| 7 | Henan Provincial People’s Hospital | Central China | Henan | Zhengzhou | Chuanyu Gao |
| 8 | Shanxi Provincial People’s Hospital | Northern China | Shanxi | Taiyuan | Chunlin Lai |
| 9 | Xinqiao Hospital, Third Military Medical University | Southwest China | Chongqing | Chongqing | Cui Bin, Lan Huang |
| 10 | China Meitan General Hospital | Northern China | Beijing | Beijing | Di Wu |
| 11 | The 309th Hospital of Chinese People’s Liberation Army | Northern China | Beijing | Beijing | Fakuan Tang, Jun Xiao |
| 12 | Zhongda Hospital, Southeast University | Eastern China | Jiangsu | Nanjing | Genshan Ma |
| 13 | The First Affiliated Hospital of Liaoning Medical University | Northeast China | Liaoning | Jinzhou | Guizhou Tao |
| 14 | Xinjiang Uygur Autonomous Region People’s Hospital | Northwest China | Xinjiang | Urumchi | Guoqing Li |
| 15 | Sir Run Run Shaw Hospital, College of Medicine, Zhejiang University | Eastern China | Zhejiang | Hangzhou | Guosheng Fu |
| 16 | Beijing Friendship Hospital, Capital Medical University | Northern China | Beijing | Beijing | Hongwei Li |
| 17 | The First Affiliated Hospital of Bengbu Medical College | Eastern China | Anhui | Bengbu | Honhju Wang |
| 18 | General Hospital of TISCO | Northern China | Shanxi | Taiyuan | Huifeng Wang |
| 19 | Dongguan People’s Hospital | Southern China | Guangdong | Dongguan | Jianfeng Ye |
| 20 | Panyu Hospital of Chinese Medicine | Southern China | Guangdong | Guangzhou | Jianhao Li |
| 21 | Peking University First Hospital | Northern China | Beijing | Beijing | Jie Jiang |
| 22 | Sun Yat-sen Memorial Hospital, Sun Yat-sen University | Southern China | Guangdong | Guangzhou | Jingfeng Wang |
| 23 | Guangdong General Hospital | Southern China | Guangdong | Guangzhou | Jiyan Chen |
| 24 | Hospital of Xinjiang Production & Construction Corps | Northwest China | Xinjiang | Urumchi | Junming Liu |
| 25 | The Military General Hospital of Beijing PLA | Northern China | Beijing | Beijing | Junxia Li |
| 26 | The First Affiliated Hospital of Guangxi Medical University | Southern China | Guangxi | Nanning | Lang Li |
| 27 | Tongren Hospital Affiliated to Shanghai Jiaotong University School of Medicine | Eastern China | Shanghai | Shanghai | Li Jiang |
| 28 | Binzou City Center Hospital | Eastern China | Shandong | Binzhou | Lijun Meng |
| 29 | The First Affiliated Hospital of Zhengzhou University | Central China | Henan | Zhengzhou | Ling Li |
| 30 | Xijing Hospital | Northwest China | Shaanxi | Xi’an | Ling Tao |
| 31 | The Affiliated Hospital of Guizhou Medical University | Southwest China | Guizhou | Guiyang | Lirong Wu |
| 32 | First Affiliated Hospital of the People’s Liberation Army General Hospital | Northern China | Beijing | Beijing | Miao Tian |
| 33 | The Second People’s Hospital of Yunnan Province | Southwest China | Yunnan | Kunming | Minghua Han |
| 34 | Haikou People’s Hospital | Southern China | Hainan | Haikou | Moshui Chen |
| 35 | Gansu Provincial Hospital | Northwest China | Gansu | Lanzhou | Ping Xie |
| 36 | The First Affiliated Hospital of Henan University of Science and Technology | Central China | Henan | Luoyang | Pingshuan Dong |
| 37 | Chenzhou First People’s Hospital | Central China | Hunan | Chenzhou | Qiaoqing Zhong |
| 38 | People’s Hospital of Qinghai Province | Northwest China | Qinghai | Xining | Rong Chang |
| 39 | Affiliated Hospital of Ningxia Medical University | Northwest China | Ningxia | Yinchuan | Shaobin Jia |
| 40 | Beijing Anzhen Hospital, Capital Medical University | Northern China | Beijing | Beijing | Shaoping Nie, Xiaohui Liu |
| 41 | North Jiangsu People’s Hospital | Eastern China | Jiangsu | Yangzhou | Shenghu He |
| 42 | Shanghai Sixth People’s Hospital | Eastern China | Shanghai | Shanghai | Shixin Ma |
| 43 | The First Hospital of Handan | Northern China | Hebei | Handan | Shuanli Xin |
| 44 | Huai’an First People’s Hospital | Eastern China | Jiangsu | Huai’an | Shuren Ma |
| 45 | The First Affiliated Hospital of Chongqing Medical University | Southwest China | Chongqing | Chongqing | Suxin Luo |
| 46 | Navy General Hospital | Northern China | Beijing | Beijing | Tianchang Li |
| 47 | Zhejiang Provincial Hospital of TCM | Eastern China | Zhejiang | Hangzhou | Wei Mao |
| 48 | The Third Xiangya Hospital of Central South University | Central China | Hunan | Changsha | Weihong Jiang |
| 49 | Affiliated Hospital of Qinghai University | Northwest China | Qinghai | Xining | Weijun Liu |
| 50 | Teda International Cardiovascular Hospital | Northern China | Tianjin | Tianjin | Wenhua Lin |
| 51 | The Second Hospital of Hebei Medical University | Northern China | Hebei | Shijiazhuang | Xianghua Fu |
| 52 | Changhai Hospital of Shanghai | Eastern China | Shanghai | Shanghai | Xianxian Zhao |
| 53 | The Second Affiliated Hospital to Nanchang University | Eastern China | Jiangxi | Nanchang | Xiaoshu Cheng |
| 54 | Hebei General Hospital | Northern China | Hebei | Shijiazhuang | Xiaoyong Qi |
| 55 | Inner Mongolia People’s Hospital | Northern China | Inner Mongolia | Hohhot | Xingsheng Zhao |
| 56 | The General Hospital of Shenyang Military Region | Northeast China | Liaoning | Shenyang | Yaling Han |
| 57 | The First Hospital of Jilin University | Northeast China | Jilin | Changchun | Yang Zheng |
| 58 | Tianjin Chest Hospital | Northern China | Tianjin | Tianjin | Yin Liu |
| 59 | Hunan Provincial People’s Hospital | Central China | Hunan | Changsha | Ying Guo |
| 60 | People’s Hospital of Yuxi City | Southwest China | Yunnan | Yuxi | Yinglu Hao |
| 61 | The People’s Hospital of Guangxi Zhuang Autonomous Region | Southern China | Guangxi | Nanning | Yingzhong Lin |
| 62 | The First Teaching Hospital of Xinjiang Medical University | Northwest China | Xinjiang | Urumchi | Yitong Ma |
| 63 | Baogang Hospital | Northern China | Inner Mongolia | Baotou | Yongdong Li |
| 64 | Tianjin Medical University General Hospital | Northern China | Tianjin | Tianjin | Yuemin Sun |
| 65 | The Second Affiliated Hospital of Zhengzhou University | Central China | Henan | Zhengzhou | Yulan Zhao |
| 66 | Nanfang Hospital of Southern Medical University | Southern China | Guangdong | Guangzhou | Yuqing Hou |
| 67 | The First Affiliated Hospital to Nanchang University | Eastern China | Jiangxi | Nanchang | Zeqi Zheng |
| 68 | The First Affiliated Hospital of Lanzhou University | Northwest China | Gansu | Lanzhou | Zheng Zhang |
| 69 | The Third Hospital of Shijiazhuang | Northern China | Hebei | Shijiazhuang | Zhenguo Ji |
| 70 | Wuxi People’s Hospital | Eastern China | Jiangsu | Wuxi | Zhenyu Yang |
| 71 | Jiangsu Province Hospital | Eastern China | Jiangsu | Nanjing | Zhijian Yang |
| 72 | The Second Hospital of Shanxi Medical University | Northern China | Shanxi | Taiyuan | Zhiming Yang |
| 73 | The Affiliated Hospital of Xuzhou Medical College | Eastern China | Jiangsu | Xuzhou | Zhirong Wang |
| 74 | Southwest Hospital, Third Military Medical University | Southwest China | Chongqing | Chongqing | Zhiyuan Song |
| 75 | The First Affiliated Hospital of Xi’an Jiaotong University | Northwest China | Shaanxi | Xi’an | Zuyi Yuan |
| 76 | Yangzhou First People’s Hospital | Eastern China | Jiangsu | Yangzhou | Aihua Li |
| 77 | Hospital 463 of Chinese People’s Liberation Army | Northeast China | Liaoning | Shenyang | Bosong Yang |
| 78 | The Central Hospital of Mianyang | Northwest China | Sichuan | Mianyang | Caidong Luo |
| 79 | Liaocheng People’s Hospital | Eastern China | Shandong | Liaocheng | Chunyan Zhang |
| 80 | Yancheng Third People’s Hospital | Eastern China | Jiangsu | Yancheng | Chunyang Wu |
| 81 | The Second Xiangya Hospital of Central South University | Central China | Hunan | Changsha | Daoquan Peng |
| 82 | The Central Hospital of Panzhihua | Northwest China | Sichuan | Panzhihua | Dawen Xu |
| 83 | The First Hospital of Qiqihar City | Northeast China | Heilongjiang | Qiqihar | Gang Xu |
| 84 | The Third the People’s Hospital of Bengbu | Eastern China | Anhui | Bengbu | Gengsheng Sang |
| 85 | The First Hospital of Jiamusi | Northeast China | Heilongjiang | Jiamusi | Guixia Zhang |
| 86 | Zhoushan People’s Hospital | Eastern China | Zhejiang | Zhoushan | Guoxiong Chen |
| 87 | Dalian Municipal Central Hospital | Northeast China | Liaoning | Dalian | Hailong Lin |
| 88 | Renmin Hospital of Wuhan University | Central China | Hubei | Wuhan | Hong Jiang |
| 89 | Ningxia People’s Hospital | Northwest China | Ningxia | Yinchuan | Hong Luan |
| 90 | The First People’s Hospital of Yunnan Province (Kunhua Hospital) | Southwest China | Yunnan | Kunming | Hong Zhang |
| 91 | The Central Hospital of Zhoukou | Central China | Henan | Zhoukou | Hualing Liu |
| 92 | Anyang District Hospital | Central China | Henan | Anyang | Hui Liu |
| 93 | Sichuan Provincial People’s Hospital | Northwest China | Sichuan | Chengdu | Jianhong Tao |
| 94 | Mudanjiang Cardiovascular Disease Hospital | Northeast China | Heilongjiang | Mudanjiang | Jianwen Liu |
| 95 | Yichang Central Hospital | Central China | Hubei | Yichang | Jiawang Ding |
| 96 | Qilu Hospital of Shandong University | Eastern China | Shandong | Jinan | Jifu Li |
| 97 | Affiliated Hospital of Jiangsu University | Eastern China | Jiangsu | Zhenjiang | Jinchuan Yan |
| 98 | The First People’s Hospital of Nanning City | Southern China | Guangxi | Nanning | Jinru Wei |
| 99 | The First Affiliated Hospital of Fujian Medical University | Eastern China | Fujian | Fuzhou | Jinzi Su |
| 100 | Chengdu Third People’s Hospital | Northwest China | Sichuan | Chengdu | Jiong Tang |
| 101 | Yantaishan hospital | Eastern China | Shandong | Yantai | Juexin Fan |
| 102 | Qingdao Municipal Hospital | Eastern China | Shandong | Qingdao | Jun Guan |
| 103 | Zhongshan Hospital Affiliated to Fudan University | Eastern China | Shanghai | Shanghai | Junbo Ge |
| 104 | Longyan First Hospital | Eastern China | Fujian | Longyan | Kaihong Chen |
| 105 | Affiliated Hospital of Guangdong Medical College | Southern China | Guangdong | Guangzhou | Keng Wu |
| 106 | Jiangxi Provincial People’s Hospital | Eastern China | Jiangxi | Nanchang | Lang Ji |
| 107 | Anhui Provincial Hospital | Eastern China | Anhui | Hefei | Likun Ma |
| 108 | Xiangtan City Central Hospital | Central China | Hunan | Xiangtan | Lilong Tang |
| 109 | The First Hospital of Haerbin City | Northeast China | Heilongjiang | Harbin | Lin Wei |
| 110 | Central Hospital Affiliated to Shenyang Medical College | Northeast China | Liaoning | Shenyang | Man Zhang, Kaiming Chen |
| 111 | The Central Hospital of Wuhan | Central China | Hubei | Wuhan | Manhua Chen |
| 112 | Hangzhou First People’s Hospital | Eastern China | Zhejiang | Hangzhou | Ningfu Wang |
| 113 | The Central Hospital of Xuzhou | Eastern China | Jiangsu | Xuzhou | Peiying Zhang |
| 114 | The Second hospital of Dalian Medical University | Northeast China | Liaoning | Dalian | Peng Qu |
| 115 | The First Affiliated Hospital of Liaoning University of Traditional Chinese Medicine | Northeast China | Liaoning | Shenyang | Ping Hou |
| 116 | Beijing Tsinghua Changgung Hospital | Northern China | Beijing | Beijing | Ping Zhang |
| 117 | Guizhou Provincial People’s Hospital | Southwest China | Guizhou | Guiyang | Qiang Wu |
| 118 | The First Affiliated Hospital of Xiamen University | Eastern China | Fujian | Xiamen | Qiang Xie |
| 119 | Quanzhou First Hospital | Eastern China | Fujian | Quanzhou | Rong Lin |
| 120 | Wuzhou People’s Hospital | Southern China | Guangxi | Wuzhou | Shaowu Ye |
| 121 | The Central Hospital of Jilin | Northeast China | Jilin | Changchun | Shuangbin Li |
| 122 | Xiangya Hospital Central South University | Central China | Hunan | Changsha | Tianlun Yang |
| 123 | Guangzhou Red Cross Hospital | Southern China | Guangdong | Guangzhou | Tongguo Wu |
| 124 | The First Affiliated Hospital of Guangzhou Medical College | Southern China | Guangdong | Guangzhou | Wei Wang |
| 125 | The First Affiliated Hospital of Wenzhou Medical University | Eastern China | Zhejiang | Wenzhou | Weijian Huang |
| 126 | The Second Affiliated Hospital of Soochow University | Eastern China | Jiangsu | Suzhou | Weiting Xu |
| 127 | Wuhan Asia Heart Hospital | Central China | Hubei | Wuhan | Xi Su |
| 128 | The First Affiliated Hospital of Soochow University | Eastern China | Jiangsu | Suzhou | Xiangjun Yang |
| 129 | Affiliated Hospital of Yan’an University | Northwest China | Shaanxi | Yan’an | Xiaochuan Ma |
| 130 | The First People’s Hospital of Jining | Eastern China | Shandong | Jining | Xiaofei Sun |
| 131 | The Central Hospital of Taiyuan | Northern China | Shanxi | Taiyuan | Xiaoping Chen |
| 132 | West China Hospital of Sichuan University | Northwest China | Sichuan | Chengdu | Xiaoping Chen |
| 133 | The Third Affiliated Hospital of Guangzhou Medical College | Southern China | Guangdong | Guangzhou | Ximing Chen |
| 134 | The First Affiliated Hospital of Wannan Medical College | Eastern China | Anhui | Wuhu | Xingsheng Tang |
| 135 | Tangdu Hospital of The Fourth Military Medical University | Northwest China | Shaanxi | Xi’an | Xue Li |
| 136 | Shanghai East Hospital Affiliated to Tongji University | Eastern China | Shanghai | Shanghai | Xuebo Liu |
| 137 | Xiamen Cardiovascular Disease Hospital | Eastern China | Fujian | Xiamen | Yan Wang |
| 138 | Zhongnan hospital of Wuhan University | Central China | Hubei | Wuhan | Yanggan Wang |
| 139 | Fujian Provincial Hospital | Eastern China | Fujian | Fuzhou | Yansong Guo |
| 140 | The First Affiliated hospital of Dalian Medical University | Northeast China | Liaoning | Dalian | Yanzong Yang |
| 141 | The First People’s Hospital of Changde | Central China | Hunan | Changde | Yi Huang |
| 142 | The First Affiliated Hospital of China Medical University | Northeast China | Liaoning | Shenyang | Yingxian Sun |
| 143 | The Fourth Affiliated Hospital of China Medical University | Northeast China | Liaoning | Shenyang | Yuanzhe Jin |
| 144 | Cangzhou Central Hospital | Northern China | Hebei | Cangzhou | Zesheng Xu |
| 145 | The Central Hospital of Shaoyang | Central China | Hunan | Shaoyang | Zewei Ouyang |
| 146 | The People’s Hospital of Liaoning Province | Northeast China | Liaoning | Shenyang | Zhanquan Li |
| 147 | The First Affiliated Hospital of Jiamusi University | Northeast China | Heilongjiang | Jiamusi | Zhaofa He |
| 148 | Tangshan Gongren Hospital | Northern China | Hebei | Tangshan | Zheng Ji |
| 149 | Huaibei Miners General Hospital | Eastern China | Anhui | Huaibei | Zhenqi Su |
| 150 | Linyi People’s Hospital | Eastern China | Shandong | Linyi | Zhihong Ou |
| 151 | Chongqing Hechuan District People’s Hospital | Southwest China | Chongqing | Chongqing | Xin Tang |
| 152 | Yuzhou City Central Hospital | Central China | Henan | Xuchang | Qinfeng Su |
| 153 | Jianshui County People’s Hospital | Southwest China | Yunnan | Honghe | Weiqing Fan |
| 154 | Dunhua City Hospital | Northeast China | Jilin | Dunhua | Fanju Meng |
| 155 | Shenyang City Electricity Central Hospital | Northeast China | Liaoning | Shenyang | Jing Xu |
| 156 | Shanghai Jingan District Shibei Hospital | Eastern China | Shanghai | Shanghai | Bin Wang |
| 157 | Beijing Fangshan District First Hospital | Northern China | Beijing | Beijing | Xuemei Peng |
| 158 | Hebei Daming County People’s Hospital | Northern China | Hebei | Handan | Haiping Guo |
| 159 | Jiangsu Binhai County People’s Hospital | Eastern China | Jiangsu | Yancheng | Yonglin Zhang |
| 160 | The First People’s Hospital of Longquanyi District | Southwest China | Sichuan | Chengdu | Wei Tuo |
| 161 | Guangxi Hengxian County People’s Hospital | Southern China | Guangxi | Nanning | Xianan Zhang |
| 162 | Hunan Changsha County First People’s Hospital | Central China | Hunan | Changsha | Siding Wang |
| 163 | People’s Hospital of Wugang | Central China | Hunan | Shaoyang | JiaoMei Yang |
| 164 | Longhui County People’s Hospital | Central China | Hunan | Shaoyang | Xiaojun Wang |
| 165 | Heilongjiang Fujin City Central Hospital | Northeast China | Heilongjiang | Jiamusi | Jiyan Yin |
| 166 | Dalian Fourth People’s Hospital | Northeast China | Liaoning | Dalian | Huifang Zhang |
| 167 | General Hospital of Guangzhou Military Command | Southern China | Guangdong | Guangzhou | Yanlie Zheng |
| 168 | The First People’s Hospital of Horqin District, Tongliao City | Northern China | Inner Mongolia | Tongliao | Junping Fang |
| 169 | Guiyang Sixth People’s Hospital | Southwest China | Guizhou | Guiyang | Kalan Luo |
| 170 | Geological Mining Hospital of Hunan Province | Central China | Hunan | Changsha | Naiyi Liang |
| 171 | Zhangzhou Municipal Hospital of Fujian Province | Eastern China | Fujian | Zhangzhou | Changyong Liu |
| 172 | Jining City Yanzhou District People’s Hospital | Eastern China | Shandong | Jining | Jian Yang |
| 173 | The People’s Hospital Feixian | Eastern China | Shandong | Linyi | Honghua Deng |
| 174 | Tangshan City Fengrun District People’s Hospital | Northern China | Hebei | Tangshan | Lin Wang |
| 175 | Qian’an People’s Hospital | Northern China | Hebei | Tangshan | Yuheng Yang |
| 176 | Yuzhong County People’s Hospital | Northwest China | Gansu | Lanzhou | Xiaowei Peng |
| 177 | Baiyin Cite Center Hospital | Northwest China | Gansu | Baiyin | Fang Zhao |
| 178 | Mingguang People’s Hospital | Eastern China | Anhui | Chuzhou | Yong Li |
| 179 | Xihua County People’s Hospital | Central China | Henan | Zhoukou | Chuntong Wang |
| 180 | Zhalantun People’s Hospital | Northern China | Inner Mongolia | Hulunbeier | Yuhua Zhu |
| 181 | Fengrun District Second People’s Hospital | Northern China | Hebei | Tangshan | Jingshan Zhao |
| 182 | Zhangping City Hospital | Eastern China | Fujian | Zhangpin | Jinxing Yi |
| 183 | Fuqing Cite Hospital | Eastern China | Fujian | Fuqing | Ping Chen |
| 184 | The Eight Affiliated Hospital, Sun Yat-sen University | Southern China | Guangdong | Guangzhou | Nan Jia |
| 185 | The Second Affiliated Hospital of Qiqihar Medical University | Northeast China | Heilongjiang | Qiqihar | Yanli Wang |
| 186 | Wuhan University of Science and Technology Hospital | Central China | Hubei | Wuhan | Jing Hu |
| 187 | Baotou City Center Hospital | Northern China | Inner Mongolia | Baotou | Ruiping Zhao |
| 188 | Shanghai Jiading District Center Hospital | Eastern China | Shanghai | Shanghai | Xia Chen |
| 189 | Datong City Second People’s Hospital | Northern China | Shanxi | Datong | Xiaoqin Zhang |
| 190 | Binyang People’s Hospital | Southern China | Guangxi | Binyang | Fudong Gan |
| 191 | Deqing People’s Hospital | Eastern China | Zhejiang | Deqing | Fangfang Huang |
| 192 | Xinmi people’s hospital | Central China | Henan | Xinmi | Xiaolei Li |
| 193 | Dongguan Changping hospital | Southern China | Guangdong | Dongguan | Haiyun Lin |
| 194 | Gongyi people’s hospital | Central China | Henan | Gongyi | Tianmin Du |
| 195 | Ye County people’s hospital | Central China | Henan | Yexian | Jie Yang |
| 196 | The second people’s hospital of Mengcheng | Eastern China | Anhui | Mengcheng | Pengfei Zhang |
| 197 | Nanpi People’s Hospital | Northern China | Hebei | Nanpi | Hui Dong |
| 198 | Shimen People’s Hospital | Central China | Hunan | Shimeng | Chuanliang Liang |
| 199 | Tieli People’s Hospital | Northeast China | Heilongjiang | Tieli | Yanbo Niu |
| 200 | Sihui People’s Hospital | Southern China | Guangdong | Sihui | Yuehua Huang |
| 201 | Chest Hospital of Xinjiang Uygur Autonomous Region | Northwest China | Xinjiang | Urumchi | Dongsheng Chai |
| 202 | Beian First People’s Hospital | Northeast China | Heilongjiang | Bei’an | Dongyan Li |
| 203 | Zunhua People’s Hospital | Northern China | Hebei | Zunhua | Xiaoli Yang |
| 204 | Lujiang People’s Hospital | Eastern China | Anhui | Lujiang | Qichun Wang |
| 205 | Qinyang People’s Hospital | Central China | Henan | Qinyang | Xiaowen Ma |
| 206 | Longmen People’s Hospital | Southern China | Guangdong | Longmen | Yingchao Luo |
| 207 | Quyang Renji Hospital | Northern China | Hebei | Quyang | Congliang Zhang |
| 208 | Nenjiang People’s Hospital | Northeast China | Heilongjiang | Nenjiang | Shuhua Zhang |
| 209 | Longjiang First People’s Hospital | Northeast China | Heilongjiang | Longjiang | Yuhuan Shi |
| 210 | Li County Hospital of Traditional Chinese Medicine | Central China | Hunan | Changde | Songbai Li |
| 211 | Luan County People’s Hospital | Northern China | Hebei | Luanxian | Guo Li |
| 212 | Yulong Hospital | Southwest China | Yunnan | Yulong | Zeyuan He |
| 213 | Huining People’s Hospital | Northwest China | Gansu | Huining | Jiabin Xi |
| 214 | Yuncheng Hospital | Eastern China | Shandong | Yuncheng | Jinglan Diao |
| 215 | Hepu People’s Hospital | Southern China | Guangxi | Hepu | Meisheng Lai |
| 216 | Duzishan Petrochemical Hospital | Northwest China | Xinjiang | Dushanzi | Shuqiu Qu |
| 217 | Guiding People’s Hospital | Southwest China | Guizhou | Guiding | Guoduo Chen |
| 218 | People’s Hospital of Rongchang District | Southwest China | Chongqing | Chongqing | Jie Chen |
| 219 | Ningbo First Hospital | Eastern China | Zhejiang | Ningbo | Huimin Chu |
| 220 | Ledong Second People’s Hospital | Southern China | Hainan | Ledong | Xiufeng Chen |
| 221 | Guang’an People’s Hospital | Southwest China | Sichuan | Guang’an | Tian Tuo |
| 222 | Linfen People’s Hospital | Northern China | Shanxi | Linfen | Junping Deng |
| 223 | People’s Hospital of Bozhou District | Southwest China | Guizhou | Zunyi | Shengyong Chen |
| 224 | Dianjiang People’s Hospital | Southwest China | Chongqing | Dianjiang | Yang Yu |
| 225 | First Affiliated Hospital of Harbin Medical University. | Northeast China | Heilongjiang | Harbin | Yue Li |
| 226 | Yiliang Hospital | Southwest China | Yunnan | Yiliang | Liqiong Yang |
| 227 | Haidong Ping’an District Hospital of Traditional Chinese Medicine | Northwest China | Qinghai | Haidong | Guoqin Xin |
| 228 | Ningjin People’s Hospital | Eastern China | Shandong | Ningjin | Tao Zhang |
| 229 | Yutian Hospital | Northern China | Hebei | Yutian | Xiaoyun Feng |
| 230 | Yanting People’s Hospital | Southwest China | Sichuan | Yanting | Mingcheng Bai |
| 231 | The Fourth Affiliated Hospital Zhejiang University School of Medicine | Eastern China | Zhejiang | Yiwu | Shudong Xia |
| 232 | Wuxi Xishan People’s Hospital | Eastern China | Jiangsu | Wuxi | Xudong Li |
| 233 | Dongfeng Hospital | Northeast China | Jilin | Dongfeng | Wei Liu |
| 234 | Zhijin People’s Hospital | Southwest China | Guizhou | Zhijin | Zhongshan Wang |
| 235 | Huaiyang People’s Hospital | Central China | Henan | Huaiyang | Li Wei |
| 236 | Suizhou Central Hospital | Central China | Hubei | Suizhou | Fengwei Li |
| 237 | Tonglu First People’s Hospital | Eastern China | Zhejiang | Tonglu | Xiaolan Li |
| 238 | Xiantao First People’s Hospital | Central China | Hubei | Xiantao | Dongmei Zhu |
| 239 | Honghu People’s Hospital | Central China | Hubei | Honghu | Hong Liu |
| 240 | Xinjin County Hospital of Traditional Chinese Medicine | Northwest China | Sichuan | Xinjin | Yingbi Su |

Table S2.Joint association of TyG index and MACEs

| Variables | Case | Adjusted HR(95%Cl) | P value | P value for interaction |
| --- | --- | --- | --- | --- |
| age |  |  |  | <0.001 |
| ≤60 | 40267 | 1.162(1.039,1.3) | 0.008 |  |
| >60 | 60846 | 1.352(1.269,1.44) | <0.001 |  |
| Sex |  |  |  | 0.001 |
| Male | 74021 | 1.267(1.183,1.356) | <0.001 |  |
| Female | 27092 | 1.366(1.244,1.499) | <0.001 |  |
| Smoking |  |  |  | 0.193 |
| No | 76333 | 1.314(1.236,1.397) | <0.001 |  |
| Yes | 24780 | 1.248(1.101,1.415) | 0.001 |  |
| Diabetes |  |  |  | <0.001 |
| No | 78255 | 1.299(1.215,1.39) | <0.001 |  |
| Yes | 22858 | 1.305(1.185,1.436) | <0.001 |  |
| Hyperlipidemia |  |  |  | 0.316 |
| No | 55808 | 1.699(1.532,1.885) | <0.001 |  |
| Yes | 45305 | 1.361(1.234,1.501) | <0.001 |  |
| Renal function |  |  |  | <0.001 |
| No | 91104 | 1.191(1.112,1.276) | <0.001 |  |
| Yes | 10009 | 1.406(1.283,1.54) | <0.001 |  |
| Hypertension |  |  |  | 0.442 |
| No | 46997 | 1.268(1.166,1.378) | <0.001 |  |
| Yes | 54116 | 1.325(1.231,1.426) | <0.001 |  |
| PCI |  |  |  | <0.001 |
| No | 92361 | 1.308(1.235,1.386) | <0.001 |  |
| Yes | 8752 | 1.262(1.036,1.538) | 0.021 |  |
